# Conceptual framework for task shifting and task sharing: an international Delphi study

**DOI:** 10.1186/s12960-021-00605-z

**Published:** 2021-05-03

**Authors:** Aaron M. Orkin, Sampreeth Rao, Jeyasakthi Venugopal, Natasha Kithulegoda, Pete Wegier, Stephen D. Ritchie, David VanderBurgh, Alexandra Martiniuk, Fabio Salamanca-Buentello, Ross Upshur

**Affiliations:** 1 Department of Family and Community Medicine, University of Toronto, Toronto, Canada; 2 Institute for Health Policy, Management, and Evaluation, Dalla Lana School of Public Health, University of Toronto, Toronto, Canada; 3 Li Ka Shing Knowledge Institute, Unity Health, Toronto, Toronto, Canada; 4 Temerty Faculty of Medicine, University of Toronto, Toronto, Canada; 5 Association of Public Health Epidemiologists of Ontario, Toronto, Canada; 6 Women’s College Institute for Health System Solutions and Virtual Care, Women’s College Hospital, Toronto, Canada; 7 Humber River Hospital, Toronto, Canada; 8 School of Kinesiology and Health Sciences, Faculty of Health, Laurentian University; Sudbury, Toronto, Canada; 9 Department of Family Medicine, McMaster University, Hamilton, Canada; 10 School of Public Health, Faculty of Medicine and Health, University of Sydney, Sydney, Australia; 11 Bridgepoint Collaboratory for Research and Innovation, Lunenfeld–Tanenbaum Research Institute, Sinai Health System, Toronto, Canada; 12 The George Institute for Global Health, Sydney, Australia

**Keywords:** Task shifting, Task sharing, Conceptual framework, Delphi process, Global health, Health human resources, Community health workers

## Abstract

**Background:**

Task shifting and sharing (TS/S) involves the redistribution of health tasks within workforces and communities. Conceptual frameworks lay out the key factors, constructs, and variables involved in a given phenomenon, as well as the relationships between those factors. Though TS/S is a leading strategy to address health worker shortages and improve access to services worldwide, a conceptual framework for this approach is lacking.

**Methods:**

We used an online Delphi process to engage an international panel of scholars with experience in knowledge synthesis concerning TS/S and develop a conceptual framework for TS/S. We invited 55 prospective panelists to participate in a series of questionnaires exploring the purpose of TS/S and the characteristics of contexts amenable to TS/S programmes. Panelist responses were analysed and integrated through an iterative process to achieve consensus on the elements included in the conceptual framework.

**Results:**

The panel achieved consensus concerning the included concepts after three Delphi rounds among 15 panelists. The COATS Framework (Concepts and Opportunities to Advance Task Shifting and Task Sharing) offers a refined definition of TS/S and a general purpose statement to guide TS/S programmes. COATS describes that opportunities for health system improvement arising from TS/S programmes depending on the implementation context, and enumerates eight necessary conditions and important considerations for implementing TS/S programmes.

**Conclusion:**

The COATS Framework offers a conceptual model for TS/S programmes. The COATS Framework is comprehensive and adaptable, and can guide refinements in policy, programme development, evaluation, and research to improve TS/S globally.

**Supplementary Information:**

The online version contains supplementary material available at 10.1186/s12960-021-00605-z.

## Background

Shortages of trained and accessible health professionals are a threat to health and health equity worldwide. Health systems and professionals face substantial burdens to respond to new pressures, such as the COVID-19 pandemic, while maintaining the operation of routine services and care. Task shifting and task sharing (TS/S) involve the strategic redistribution of tasks among health workforce teams and personnel. Specific tasks are moved, shared or delegated, usually from highly trained health workers to those with shorter training or fewer qualifications, including laypeople [1].

Task shifting and task sharing is a promising way to address global health workforce shortages and insufficient access to care for critical health problems, and has been used effectively to improve health in a range of contexts and conditions [1, 2]. Task shifting and task sharing strategies allow for more efficient use of health human resources, especially as health systems worldwide struggle to maintain essential services while responding to the COVID-19 pandemic [2].

Conceptual frameworks lay out the key factors, constructs, and variables involved in a given phenomenon, as well as the relationships between those factors [3]. Conceptual frameworks can guide programme design and implementation, and assessments of programme performance, including effectiveness, equity, efficiency and quality [3, 4]. Conceptual frameworks offer a theoretical synthesis of a given phenomenon, however, there are no conceptual frameworks to identify commonalities among the contexts and conditions suited to TS/S programmes [5, 6]. A conceptual framework for the conditions and contexts suited to TS/S programmes could refine the development and implementation of TS/S programmes and research globally.

The goal of this study was to develop a conceptual framework for TS/S, using an online Delphi process to engage an international panel of scholars with experience in knowledge synthesis around TS/S.

## Methods

### Delphi design

We used a Delphi process to guide the iterative development of the Concepts and Opportunities to Advance Task Shifting and Task Sharing (COATS) Framework [7]. We used a modified online Delphi process to engage and gather opinions from scholarly experts on TS/S, and identify consensus responses to two research questions:

1. What differentiates task shifting from other circumstances where workers with limited training or qualifications are involved in health care?
2. What criteria can be used to identify conditions and contexts suited to task shifting?

The Delphi method is a structured approach to develop consensus among experts through a series of questionnaires and structured feedback. It involves collecting and distilling knowledge from a dispersed panel of experts to build reliable group consensus on a specific issue. This approach has been used to forecast trends across a range of policy and research domains, and to drive decision-making and resource allocation [8]. There are numerous Delphi variants, but most share design features such as purposive sampling, participant anonymity, iterative design, structured communication between participants and researchers, thematic analysis of expert responses, and statistical aggregation of group feedback [7, 9, 10]. Delphi methods add structure to the process of building group consensus and reduce biases that can arise in face-to-face meetings by limiting the social, psychological, and political aspects of conventional group interactions, improving the quality, efficiency, long-term accuracy and validity of panelists’ predictions and judgements [10, 11].

The Delphi panel and method were not intended to deliver consensus or final approval for the specific language, structure, and format of the conceptual framework. The Delphi process was intended to solicit and synthesize a broad range of knowledge and experience concerning TS/S and provide consensus on the elements included in the COATS Framework.

### Panel selection, recruitment and retention

We determined that three Delphi rounds using email for correspondence would be sufficient to achieve consensus and stability within our expert panel [7, 9, 10]. We aimed to retain a minimum of 12 experts after three rounds of Delphi participation, and based on our experience with previous Delphi studies, we planned for 40% attrition in each round. Therefore, we estimated that 40 invitees would be required in the first round of the Delphi.

We used two techniques to convene the panel of scholarly experts. The first set of panelists recruited to the Delphi were the corresponding authors from published, international systematic reviews on TS/S (n = 36). Rather than approaching researchers engaged in the evaluation of individual TS/S programmes, we chose scholars who had demonstrated experience in knowledge synthesis around TS/S and could contribute to the development of a conceptual framework. We deliberately sought scholars who had published systematic reviews because these experts would have high-level, global knowledge of TS/S and the concepts needed to synthesize multiple TS/S programmes. They were identified from a separate systematic review underway by members of our research team [12]. Given that these invitees were corresponding authors on global systematic reviews concerning TS/S, this sample offered a panel with global and representative knowledge of the scholarly literature and research concerning TS/S. Second, panelists in our initial set of 36 contacts were invited to recommend colleagues who, in their opinion, might be interested in participating in this Delphi on the basis of their work or expertise.

Throughout the Delphi process, panelists were blinded to the identity of the other panelists, except for the individuals who referred us to subsequent panelists. Survey content was never associated with a panelist identifier; only the investigators could associate panelists with responses. All the included questions from the survey concerned the respondents’ area of professional expertise.

We sent email reminders to panelists on a weekly basis to increase the response rate. As an incentive to complete the study and to express appreciation for panelists’ participation, we offered to donate in the participant’s name to a medical humanitarian organization of the participants’ choice. We gathered information about panelists’ institutional affiliations and scholarly work from their publicly available academic profiles and publications.

### Delphi rounds, data collection and analysis

To ensure a systematic and meaningful synthesis of responses, we drafted and refined the questions asked of the panelists in every round. We piloted the questionnaires among members of our authorship team who were not directly involved in designing the Delphi. We communicated with the panelists in English and used Google Forms to conduct our surveys.

We developed a prespecified definition of consensus based on two criteria [13]. First, the panel was eligible to have achieved consensus around a given survey item if at least 70% of respondents agreed with that item. When using a seven-point Likert scale, we defined “disagreement” as a score of three or less. This criterion ensured that a strong majority of respondents agreed with any included survey item. Second, an item was said to have achieved consensus only if none of the dissenting respondents raised concerns that were fundamentally incompatible with the inclusion of that survey item. This criterion aligns with approaches from formal consensus decision-making, where a structured discussion is used to understand and resolve the merits and drawbacks of a given proposal [14]. This approach recognizes that essential insights can be tendered by a minority of decision-makers, and attends to the substance of minority opinions. Procedurally, these minority opinions were gathered by requiring that panelists offer free-text comments if they disapproved of a survey item. We analysed these free-text responses and incorporated that feedback into subsequent rounds of the Delphi and into the final COATS framework. As the analysis advanced, an emphasis on and reiteration of certain issues above others became more apparent. These elements coalesced into the final categories and themes.

Round I was designed to elicit broad and general concepts from the panelists using unstructured, open-ended, questions:

1. What is, in your opinion, the purpose of task shifting?
2. What are the three to five characteristics of a health problem that make it amenable to task shifting?
3. What are three to five examples of delegation of responsibilities from highly qualified health workers to individuals with fewer qualifications and shorter training that are not task shifting?

Following Round I, investigators combined and analysed the panelists’ responses in a taxonomy according to common themes and categories. We attempted to make the items on each list mutually exclusive and comprehensive. We synthesized these findings in a survey to elicit participants’ level of agreement with each of the themes and categories on a seven-point Likert scale for Round II. This survey also offered free-text response options for panelists to add additional comments or categories as required.

Our study initially used the term "task shifting" in isolation, rather than the broader concepts of both task shifting and task sharing. In Round I, panelists raised conceptual distinctions between task shifting and task sharing. Recognizing that this had emerged organically from the Delphi process, we added questions to refine the distinction between task shifting and task sharing and integrated this distinction into our conceptual model.

Once we received all responses from Round II, these data were again reviewed by the investigators. Concepts were eliminated and retained on the basis of the panelists’ scores and collapsed into more general categories, including a definition of TS/S, the purpose of TS/S, opportunities arising from TS/S programmes, and conditions required for the implementation of TS/S. These results were sent back to the panelists as a survey for Round III. Panelists were asked to review the final list of items, state whether they agreed or disagreed with each item, and voice concerns or comments in free text. Following Round III, the investigators integrated the experts’ consensus responses into a reasonable and manageable set of concepts and sub-concepts to form the conceptual framework [3].

## Results

The Delphi process was initiated on August 26, 2019 and completed on June 20, 2020. The process was paused in March through May 2020 given the COVID-19 pandemic.

**Fig. 1:**
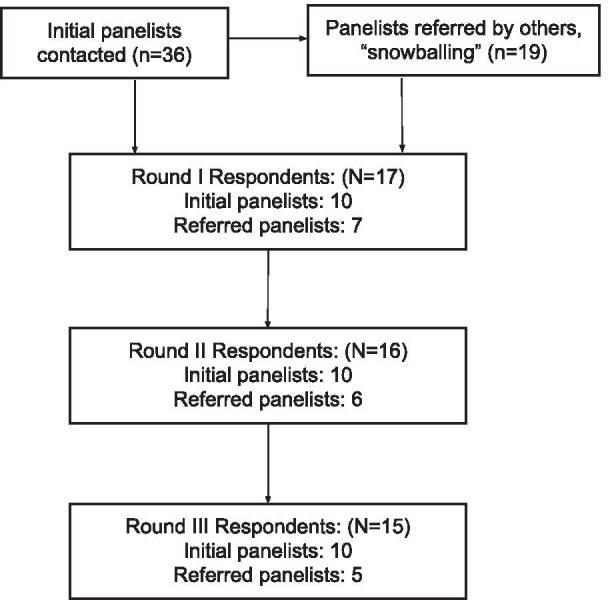
Flow chart of panelist recruitment and retention

Figure 1 provides a flowchart of panelist recruitment and retention in each of the Delphi phases. Although the response rate for the first Delphi round was 31% (17/55), attrition thereafter was low, with one non-responding panelist in each of the subsequent rounds. Fifteen (15) panelists participated in the full Delphi. All panelists were university faculty or research institute appointees with expertise related to TS/S in the United States (7), South Africa (3), the United Kingdom (2), Nigeria (1), India (1), and Australia (1). Panelists included qualitative and quantitative researchers with primary appointments in public health and epidemiology (6), medicine (3), medical anthropology (3), clinical psychology (2), and health economics (1). Twelve panelists conducted all or most of their work concerning TS/S in low- or middle-income countries.

The panel arrived at a consensus concerning the themes and content of the conceptual model following three Delphi rounds. Additional file 1 provides the questionnaires for each round of the Delphi with summary statistics of panelist responses to each item. In Round I, 17 concepts were identified to describe conditions and contexts suited to TS/S. In Round II, these features were distilled into eight conditions by combining similarly ranked survey items and removing lower ranked features. In Round III, the eight conditions were split into four necessary conditions and four important considerations based on dissenting participants’ reasoning. Additional file 1 provides an analysis of dissenting views from Round III and how these were interpreted and incorporated into the COATS Framework. Dissenting views did not tend to oppose the concepts in the emerging framework. Dissenting comments generally offered feedback to soften or adapt the emerging framework so that it would be applicable to a wider range of contexts, communities of practice, and specific circumstances. Round III achieved consensus around all survey items.

**Fig. 2:**
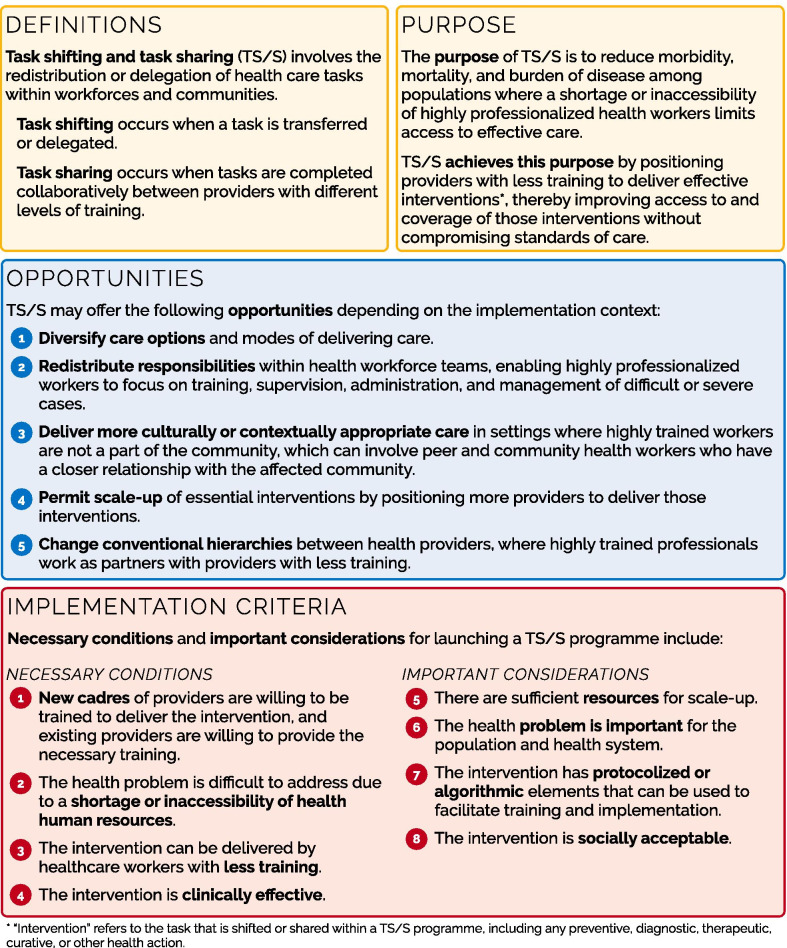
Concepts and Opportunities to Advance Task Shifting and Task Sharing (COATS) Framework

The COATS Framework consists of three elements (Fig. 2):

1. Definition and purpose of TS/S programmes (Yellow Box);
2. Opportunities arising from TS/S programmes (Blue Box); and
3. Criteria for the implementation of a TS/S programme (Red Box).
  The analysis below describes how these elements emerged from the Delphi process and how the framework can be implemented and adapted for various contexts.
  Throughout the COATS framework, the term “intervention” is used to refer to the task that is shifted or shared in the context of a TS/S programme. Interventions in this context may be preventive, curative, therapeutic, diagnostic, or another health action. For linguistic clarity, we recommend that the overall TS/S initiative be referred to as a programme rather than an intervention.
  1. *Definition and purpose of TS/S sharing programmes (Yellow Box)*
    The COATS Framework expands on the dominant WHO definition of task shifting with a revised general definition and purpose statement for TS/S programmes. Where the WHO defines task shifting as the “rational redistribution of tasks among health workforce teams”, the COATS definition offers a broader concept involving the redistribution or delegation of health care tasks within workforces or communities. This reference to communities underscores the role of lay and informal providers in TS/S initiatives.
    Delphi panelists offered feedback concerning the distinction between task shifting and task sharing. This led to refinements in the COATS Framework to offer distinctions between task shifting and task sharing along with an overarching definition of TS/S. This distinction is also reflected in the TS/S literature [15–17]. In Round II, seven panelists identified task shifting and task sharing as related but different entities, where task shifting concerns delegation, while task sharing focuses on collaboration between different workers. Another eight panelists indicated that task shifting and task sharing refer to the same phenomenon, but that task sharing is the preferred term because it captures the intrinsically collaborative nature of these undertakings. The COATS Framework captures this distinction, and permits programme developers, scholars and other stakeholders to make deliberate decisions regarding their use of task shifting versus task sharing terminology and approaches in their own work.
    The generalized purpose statement indicates that TS/S is intended to reduce morbidity, mortality, and burden of disease among populations where the inaccessibility of professionalized cadres limits access to effective care. Although health care tasks might be redistributed to providers with less training to deliver cost savings or improve operational efficiencies, this purpose statement reinforces that TS/S occurs when health care redistribution and delegation is driven by efforts to enhance health equity by meeting the health needs of underserved populations [1, 18, 19]. The general purpose statement also asserts that TS/S achieves its goals without compromising the standards of care, including maintaining safety and quality [20–23].
  2. *Opportunities arising from TS/S programmes (Blue Box)*
    The COATS Framework offers five opportunities for TS/S programmes to deliver health system improvements, depending on the implementation context. The Delphi panelists identified that TS/S programmes are diverse and contextual, and that a given programme may therefore present opportunities that are particular to their specific settings and circumstances.
    These five opportunities demonstrate that TS/S programmes may have strategic functions that extend beyond reductions in morbidity and mortality. A given TS/S programme may build on one or more of these opportunities—or none at all. For example, a mental health TS/S programme may be designed specifically to deliver more culturally appropriate care by engaging community members who are more likely than professionals to share cultural, linguistic and social background with those receiving care [24]. Other TS/S initiatives, such as peer worker engagement in harm reduction programmes, may emphasize a change to conventional professional hierarchies by positioning peer workers to assume roles and responsibilities within the healthcare system that were previously restricted to professionals [25]. The rapid scale-up of essential interventions has been a priority for TS/S programmes related to HIV care in sub-Saharan Africa [21, 26]. Practitioners, programme developers, policymakers and researchers may use these five opportunities to plan and appraise specific TS/S programmes within their specific contexts and programme goals.
  3. *Implementation criteria for TS/S programmes (Red Box)*

The final section of the COATS Framework provides criteria for programme developers, policymakers, researchers and other stakeholders to assess if a given condition and context is suited to TS/S. Panelists identified that conditions that are critical to the success of TS/S programmes in one setting may be less relevant or absent in others.

The criteria for the implementation of a TS/S programme are divided into “necessary conditions” and “important considerations”. Necessary conditions refer to features of the available workers, the health problem, and the intervention that will generally need to be in place to make a TS/S programme successful. For example, workers willing to be trained to deliver the intervention and workers willing to provide that training will both be prerequisite for any successful TS/S programme, and programme developers should consider these needs as they conceive of a new TS/S initiative. Important considerations refer to concepts that will enable success in some settings but may be less relevant in others. For example, one panelist indicated that TS/S often requires resources for scale-up, but that “those resources don’t always need to be identified before the intervention is developed or tested.” Another panelist identified that the social acceptability of an intervention may not be a precondition to a successful TS/S programme because TS/S may be used to help improve the social acceptability of an intervention within a given community (Additional file 1).

The framework does not prescribe a measure or threshold to establish if the criteria have been met. These criteria are intended for interpretation and adaption to their specific context so that practitioners and programme developers can appraise whether and how the criteria have been addressed in their particular circumstances.

## Discussion

Task shifting and task sharing is a leading and promising health systems strategy to address health workforce shortages, transform health care delivery, and improve health outcomes and inequities [1, 2]. However, TS/S is not a universal solution to insufficient or inequitable access to care. Conceptual frameworks can guide and refine a range of public health policies and decision-making, including for example, the implementation of screening programmes, immunization programmes, action on the social determinants of health, or public health ethics [4, 27–30]. The COATS Framework offers a conceptual framework to guide and refine TS/S programmes.

Other researchers have examined the various context-specific facilitators and barriers to successful TS/S implementation [31, 32]. This study builds on this research with a consensus from international TS/S scholars to advance a conceptual framework for TS/S programmes. A central strength of our study is the use of a high quality international Delphi process, with reproducible panel selection processes, prespecified consensus criteria, and a prespecified number of Delphi rounds [13]. This process supported the iterative theorization processes involved in the development of a conceptual framework, achieved consensus across the COATS concepts, and integrated the breadth and diversity of TS/S experience and expertise worldwide [3]. The framework was built based on the prevailing consensus of the panelists, but also incorporated dissenting views to ensure that the resulting framework incorporated varied terminology (task shifting and task sharing) and was adaptable to a wide range of contexts and circumstances.

The COATS Framework offers a practical definition of TS/S (including the distinction between task shifting and task sharing approaches), and guides programme development and implementation with a central purpose statement for TS/S. The necessary conditions and important considerations provides criteria that stakeholders can use to support TS/S programme implementation. The COATS Framework is both simple and adaptable: it offers a singular purpose and conditions suitable to all TS/S programmes, but also incorporates opportunities and criteria that attend to the diversity of circumstances or contexts where TS/S may be implemented. The COATS Framework is intended to prompt and support adaptations to local circumstances and contexts.

Our study and resulting framework have limitations. Our Delphi panel was convened deliberately to engage panelists with scholarly experience in conducting syntheses across TS/S programs and elicit consensus among these practitioners. The findings are therefore a consensus among participating panelists and may not be representative of other TS/S experts. One-third of panelists were from low- and middle-income countries and the majority conducted their work concerning TS/S in lower-income populations. Therefore, our findings and framework may require adaptation for other populations and settings. Panelists’ expertise may differ from the voices of policymakers, providers, or patients, and the resulting framework may well be adapted by other stakeholders. Our definition of TS/S did not explicitly include task redistribution directly to patients or artificial intelligence systems [6]. However, patient self-care and artificial intelligence may be sufficiently distinct from conventional TS/S to merit separate conceptual frameworks.

## Conclusion

The COATS Framework delivers an essential theorization of TS/S. Better theorization of TS/S can drive more deliberate, strategic, and effective programmes. The COATS Framework is both comprehensive and adaptable, and suited to support refinements across a range of TS/S policy and programmes, including planning, decision-making, implementation, evaluation and research worldwide.

## Supplementary Information

**Additional file 1.** TS/S Delphi Questionnaires and Analysis of Dissenting Views.

## Data Availability

All data generated or analysed during this study are included in this published article [and its additional files].
