# Supplementary material for: Conceptual framework for task shifting and task sharing: an international Delphi study
Source: Hum Resour Health. 2021 May 3;19:61. doi: 10.1186/s12960-021-00605-z (PMC8091141; doi:10.1186/s12960-021-00605-z)
Supplement: Supplementary file 1 — Additional file 1. TS/S Delphi Questionnaires and Analysis of Dissenting Views. [file 12960_2021_605_MOESM1_ESM.docx]

# Supplement A: TS/S Delphi Questionnaires

| **Table SA-1: Round II Questionnaire and Summary Results** | | |
| --- | --- | --- |
| **Question 1,** *Purpose of task shifting: What is the purpose of task shifting?* (Likert from 1-7, 1=Strongly disagree; 7=Strongly agree) | **Median Score (N=16)** | **Rank*** |
| The purpose of task shifting is to reduce morbidity, mortality and disease prevalence among populations where shortages of highly professionalized health care providers limits access to effective interventions. | 7 | 1 |
| The purpose of task shifting is to provide health care that is more culturally appropriate and equitably accessible than could be provided by highly professionalized health care providers who are not part of the community. | 6 | 4 |
| The purpose of task shifting is to facilitate the involvement of peer or community health workers, who may have closer relationships with affected patients or communities. | 5 | 6 |
| The purpose of task shifting is to improve access to and coverage of effective health care interventions in low-resource settings, without compromising standards of care. | 6 | 2 |
| The purpose of task shifting is to diversify care options and modes of delivering specific interventions. | 5 | 7 |
| The purpose of task shifting is to address health human resources shortages by positioning providers with less training to deliver effective interventions. | 6 | 3 |
| The purpose of task shifting is to deliver superior care for complex health problems by diversifying the care team. | 4 | 11 |
| The purpose of task shifting is to scale-up interventions rapidly by training large numbers of providers to deliver those interventions. | 5 | 8 |
| The purpose of task shifting is to distribute responsibilities within health workforce teams, enabling highly professionalized workers to focus on training, supervision, administration, and management of difficult or severe cases. | 5 | 5 |
| The purpose of task shifting is to reshape the way that health systems are designed and organized, and the way health care duties, responsibilities, and authority for care are allocated. | 4 | 9 |
| The purpose of task shifting is to change conventional hierarchical relationships between the providers who deliver care so that highly trained professionals work as partners with providers with less training. | 4 | 10 |
| Are there any other essential purposes of task shifting that, in your opinion, should be added to this list? If so, please write these purposes in the text box below. Otherwise, simply write “None”. | N/A | N/A |
| **Question 2, Task shifting and Task Sharing:**  *Please choose the statement below that, in your opinion, best characterizes the relationship between task shifting and task sharing.* | **n** | **Rank*** |
| 1. "Task Shifting" and "Task Sharing" are synonyms that refer to the same processes in health systems. | 0 | 4 |
| 1. Task shifting and task sharing refer to the same phenomenon, but task sharing is the preferred term because it better captures the collaborative nature of these undertakings. | 8 | 1 |
| 1. Task sharing is a type of task shifting, where greater emphasis is placed on sharing responsibility for a given task between providers with different training within the health care team. | 3 | 2 |
| 1. Task shifting and task sharing are related but distinct entities. In task shifting, highly trained providers transfer tasks to providers with less training. In task sharing, a new team is formed and tasks are completed collaboratively. | 3 | 2 |
| Other (with comments)   - *“I don’t know”* - *“I agree that sometimes task sharing is both option 3 and 4. my preferred form would be for all task shifting to be task sharing but in reality the two are distinct.”* | 2 | 3 |
| **Question 3, Conditions Suited to Task Shifting:** *In your opinion, how important are the following characteristics to make a health problem amenable to task shifting?* (Likert from 1-7, 1=not important; 7=extremely important) | **Median Score** | **Rank*** |
| The condition represents a considerable burden for the health system. | 5 | 10 |
| The condition is highly prevalent. | 4 | 12 |
| The condition has been difficult to address due to a shortage of available health services. | 7 | 1 |
| The condition can be identified through active, simple screening. | 5 | 10 |
| The condition can be evaluated and diagnosed with simple instruments and without the need for advanced technical skills. | 5 | 8 |
| The condition requires frequent monitoring and follow-up. | 5 | 11 |
| The condition is therapeutically complex. | 3 | 14 |
| The condition is socially complex. | 4 | 13 |
| The condition affects an underserved population or community. | 5 | 9 |
| The treatment is clinically effective. | 6 | 5 |
| The treatment has a protocolized or algorithmic aspect that facilitates decision-making. | 6 | 6 |
| The treatment requires a team for implementation. | 5 | 13 |
| The treatment is socially acceptable. | 5 | 9 |
| Treatment by non-specialist health care workers is socially acceptable. | 6 | 3 |
| Adequate resources exist to scale-up the treatment. | 6 | 7 |
| New cadres of providers are willing to be trained to take on new health care tasks. | 6 | 2 |
| Existing experienced professionals are willing to train and supervise non-specialists. | 6 | 4 |
| In your opinion, are there any other features or characteristics of a health problem that would make it suitable for task shifting? If so, please write them in the text box below. Otherwise, write “None”. | N/A | N/A |
| *As computed based on the sum of scores. | | |

| **Table SA-2: Round III Questionnaire and Summary Results** | |
| --- | --- |
| **Question 1, Purpose and Opportunities for Task Shifting:**  *Please indicate if you can approve each statement below. If you do not approve of any of the statements, please indicate why in the space provided after the statements.* (Approve / Do Not Approve) | **Approvals (N=15)** |
| The 𝗣𝗨𝗥𝗣𝗢𝗦𝗘 of task shifting is to reduce morbidity, mortality and disease prevalence among populations where shortages of highly professionalized health care workers limits access to effective interventions. Task shifting achieves this purpose by positioning providers with less training to deliver effective interventions, thereby improving access to and coverage of those interventions without compromising standards of care. | 12 |
| *Task shifting also offers the following 𝗢𝗣𝗣𝗢𝗥𝗧𝗨𝗡𝗜𝗧𝗜𝗘𝗦 depending on the context where it is implemented. Task shifting can:* | **Approvals** |
| Deliver care that is more culturally or contextually appropriate in settings where highly trained workers are not a part of the community, including by involving peer and community health workers who have a closer relationship with the affected community. | 14 |
| Diversify care options and modes of delivering specific interventions. | 15 |
| Permit rapid scale-up of essential interventions by positioning large numbers of providers to deliver those interventions. | 14 |
| Change conventional hierarchies between health providers, where highly trained professionals work as partners with providers with less training. | 14 |
| Redistribute responsibilities within health workforce teams, enabling highly professionalized workers to focus on training, supervision, administration, and management of difficult or severe cases. | 15 |
| If you did not approve any of the above statements, please indicate why. | N/A |
| **Question 2, Essential Conditions:**  *The essential conditions required to launch a task shifting intervention include.*  (Approve / Do not approve) | **Approvals** |
| The health problem is important for the population and the health system. | 12 |
| The health problem is difficult to address due to a shortage or inaccessibility of health human resources. | 13 |
| The treatment can be delivered by healthcare workers with less training. | 14 |
| The treatment is clinically effective. | 14 |
| The treatment has protocolized or algorithmic elements that can be used to facilitate training and implementation. | 12 |
| The treatment is socially acceptable. | 12 |
| New cadres of providers are willing to be trained to deliver the intervention, and existing providers are willing to provide the necessary training. | 15 |
| There are sufficient resources for scale-up. | 12 |
| If you did not approve any of the above statements, please indicate why. | N/A |
| **Question 3, Task Shifting and Task Sharing:**  *Roughly half of the panelists find a difference between the terms "task shifting" and "task sharing", while the other half consider these terms synonymous. We ask that you consider the following statement and indicate if you approve or do not approve. If you do not approve, please indicate why in the space provided after the statement.* | **Approvals** |
| Task shifting occurs when a task is transferred to a health worker cadre with less training. Task sharing occurs when a new health team is formed and tasks are completed collaboratively. | 10 |

# Supplement B: Analysis of Dissenting Views

| **Table SB-1: Round III Free-Text Responses** | |
| --- | --- |
| The 𝗣𝗨𝗥𝗣𝗢𝗦𝗘 of task shifting is to reduce morbidity, mortality and disease prevalence among populations where shortages of highly professionalized health care workers limits access to effective interventions. Task shifting achieves this purpose by positioning providers with less training to deliver effective interventions, thereby improving access to and coverage of those interventions without compromising standards of care. | |
| **Responses** | **Investigator Analysis** |
| The description is great. However the terminology is a problem (most broadly and globally). I would still rather see the term “task sharing” approved within/alongside this term of “task shifting” or replace it, as often what makes the task shifting effective is that it is somewhat shared or at least supervised by specialists. it is not just a one way shift with no support - that tends to not be effective. | “TS/S” terminology adopted to ensure that the conceptual framework addresses both shifting and sharing. |
| This definition has a narrow focus on task shifting as moving 'down' a hierarchy of training, and only being in response to a shortage of 'highly professionalized health care workers'. This is a common scenario but it feels too restrictive to make it the definition. The opportunities listed below, for example, might instead be core purposes in some situations. | “TS/S” terminology as above. Remove “less training” and the notion that delegation must go ‘down’ a hierarchy of training throughout. |
| Even in areas where there are a higher number of highly trained individuals, task sharing splits up tasks among a team so that each can work to the highest scope of practice and support outreach. Often when these tasks all fall on one individual (e.g., a PCP or psychiatrist) they do not have capacity for the admin and outreach roles that improve care for the population. Regular monitoring of depression symptoms for example is not something a psychiatrist has time to monitor at a population level. | “TS/S” terminology as above. Modify “shortages” so that the purpose is not exclusively about the number of highly trained individuals. |
| *Task shifting also offers the following 𝗢𝗣𝗣𝗢𝗥𝗧𝗨𝗡𝗜𝗧𝗜𝗘𝗦 depending on the context where it is implemented. Task shifting can:*   - Deliver care that is more culturally or contextually appropriate in settings where highly trained workers are not a part of the community, including by involving peer and community health workers who have a closer relationship with the affected community. - Diversify care options and modes of delivering specific interventions. - Permit rapid scale-up of essential interventions by positioning large numbers of providers to deliver those interventions. - Change conventional hierarchies between health providers, where highly trained professionals work as partners with providers with less training. - Redistribute responsibilities within health workforce teams, enabling highly professionalized workers to focus on training, supervision, administration, and management of difficult or severe cases. | |
| **Responses** | **Investigator Analysis** |
| Having a larger workforce that requires supervision and support does not necessarily coincide with a rapid implementation. Seems they have the potential to reach more but that care and support are needed - which may not be rapid - to achieve comparable or better results compared to a smaller team of more highly trained individuals. The challenges with task sharing is that the work is dispersed so a workflow needs to be developed with regular communication and roles need to be navigated. This can take some time. On the hierarchies, it is not clear that hierarchies will be challenged and will likely continue to operate in these teams as this is so ingrained in health care delivery. | “Opportunities” need not be realized in all TS/S cases. |
| Peer-led interventions may not be considered as task shifting interventions. | “Opportunities” need not be realized in all TS/S cases. |
| Lesser trained staff may be given more responsibility (eg initiating and maintaining treatment) but I am not clear that this represents a change in 'conventional hierarchies'. | “Opportunities” need not be realized in all TS/S cases. |
| **Question 2, Essential Conditions:**  *The essential conditions required to launch a task shifting intervention include.*   - The health problem is important for the population and the health system. - The health problem is difficult to address due to a shortage or inaccessibility of health human resources. - The treatment can be delivered by healthcare workers with less training. - The treatment is clinically effective. - The treatment has protocolized or algorithmic elements that can be used to facilitate training and implementation. - The treatment is socially acceptable. - New cadres of providers are willing to be trained to deliver the intervention, and existing providers are willing to provide the necessary training. - There are sufficient resources for scale-up. | |
| **Responses** | **Investigator Analysis** |
| Some treatments (e.g., EBP delivered within needle exchange locations) may not be socially acceptable but can be task shared. This may occur with partial lack of awareness within the community of the program and also with support of local champions. | Split “essential conditions” into “necessary conditions” and “important considerations” |
| I don't think it's essential that the problem is necessarily viewed as important for "the health system." There are so many people who have opinions on what it is important at that level that I'd take that out of this statement. For the final one, I don't think having resources all figured out at the beginning of developing a task shifting intervention is absolutely essential. It needs to be designed in a way that it will be feasible to scale, but those resources don't always need to be identified before the intervention is developed or tested. | Split “essential conditions” into “necessary conditions” and “important considerations” |
| I do not see that level of importance of the health problem is an essential driver of whether a task can be shifted. If a decision has already been taken to direct health resources to a problem that is not important for the population, then task shifting should not be excluded | Split “essential conditions” into “necessary conditions” and “important considerations” |
| I agree that the treatment should be clinically effective, insofar as I would hope there is little support for propagating ineffective treatments; however, task shifting isn't limited to treatment | Split “essential conditions” into “necessary conditions” and “important considerations” |
| I do not see 'social acceptability' as a precondition for task shifting. Same logic as the first response (ie if the decision has already been taken to provide a treatment despite limited social acceptability, then task shifting should be able to be considered) | Split “essential conditions” into “necessary conditions” and “important considerations” |
| It depends how "important" is defined. Some things like mental healthcare are often very under-prioritized by communities and healthcare systems (i.e. not important if we're talking about perceptions) but are a significant aspect of morbidity, economic impact, etc (i.e. important if we're talking about disease burden). | Split “essential conditions” into “necessary conditions” and “important considerations” |
| For the others, I somewhat reluctantly disagreed with them as "essential conditions" because I thought they could be added or developed as part of the process of beginning the intervention. Of course, ideally all of these conditions would be the case for a successful program. | Split “essential conditions” into “necessary conditions” and “important considerations” |
| My concern is with the word "essential". I am not sure what health problems are not important (perhaps less important would be a better term), but I see no reason why you couldn't implement task shifting with less important health issues if it might free up resources for providers to work on other, perhaps more important health issues? Similarly, if you can implement a small-scale task-shifting project, even if there aren't resources for scale-up, why not do it? Seems like these might all be nice to have, but I'm not sure all are absolutely essential. | Split “essential conditions” into “necessary conditions” and “important considerations” |
| **Question 3, Task Shifting and Task Sharing:**  Task shifting occurs when a task is transferred to a health worker cadre with less training. Task sharing occurs when a new health team is formed and tasks are completed collaboratively. |  |
| **Responses** | **Investigator Analysis** |
| The person transferring these tasks is still training, supervising and supporting so it is still a team effort. | Does not dispute that there exists a distinction between “task shifting” and “task sharing”. |
| I wouldn't think that a task sharing intervention necessarily requires forming a new team. It could be reallocation of tasks on an existing team such that more of the tasks/responsibilities are done by someone with less training but still in collaboration with the more highly trained professional. | Remove the notion of “new team”, emphasizing instead the collaborative nature of task sharing. |
| Original objection to the more to less training framing. And it seems like one can share tasks within old teams and old protocols. | Remove the notion of “new team”, emphasizing instead the collaborative nature of task sharing. Remove concept of shifting “down” a hierarchy. |
| Might not necessarily involve the creation of a "new" health team, but may involve "redistribution" of tasks among existing/current team members. Hence, I actually prefer the term "task redistribution" | Remove the notion of “new team”, emphasizing instead the collaborative nature of task sharing. |
| Task sharing implied that the task is shifted AND there is appropriate supervision and support, and proactive link to specialist or experienced providers. | Respondent argues that all effective delegation involves support and supervision and is therefore “sharing”. Retain “shifting” alongside “sharing” (TS/S) as it remains common in the policy and practice literature. |
